# Conformational changes involved in sGC activation

**DOI:** 10.1186/2050-6511-14-S1-O12

**Published:** 2013-08-29

**Authors:** Michael A Marletta, Eric Underbakke, Melody G Campbell, Bridget Carragher, Clinton S Potter

**Affiliations:** 1 Department of Chemistry, The Scripps Research Institute, La Jolla, California 92037, USA; 2 Department of Integrative Structural and Computational Biology, The Scripps Research Institute, La Jolla, California 92037, USA

## Background

Soluble guanylate cyclase (sGC) is a central target of nitric oxide (NO) action. sGC is a heterodimeric hemoprotein. The ferrous heme efficiently traps NO and plays an intimate role in activation of the enzyme to catalyse the conversion of GTP to cGMP. sGC is also the target for cardiovascular therapies involving small molecules that stimulate sGC directly or activate oxidized or apo sGC. Efforts to fully characterize sGC catalysis have been hampered by the lack of structural information. High-resolution structures of sGC fragments (domains) have been determined and have provided important detail; however, a structure of the full-length protein, an essential piece of the puzzle, remains unsolved.

## Results

Using hydrogen-deuterium exchange-mass spectrometry (HDX-MS), higher order domain interactions have been mapped [1]. HDX-MS revealed direct interactions between the PAS domain and the heme-associated signaling helix of the H-NOX domain. Furthermore, interfaces between the H-NOX and catalytic domains were mapped using domain truncations and full-length sGC. The H-NOX domain buries surfaces of the α1 catalytic domain proximal to the cyclase active site, suggesting a signaling mechanism involving NO-induced de-repression of catalytic activity. This method is now being extended to map the conformational changes that take place in sGC with NO binding and other small molecules that influence catalytic activity. In addition, significant advances toward a full-length structure have been obtained using single-particle electron microscopy.

**Figure 1:**
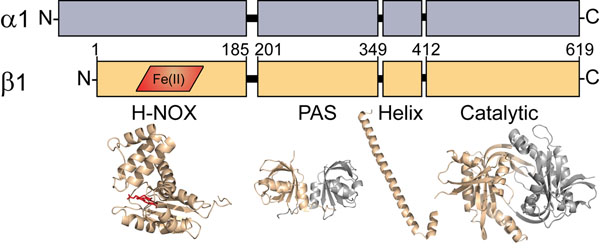
**Domain organization of sGC subunits** Each sGC subunit, α1 (grey) and β1 (tan), is composed of four modular domains. The H-NOX domain of the β1 subunit contains the heme co-factor (red). Amino acid numberings approximate the boundaries of the β1 domains. The H-NOX structure is modeled from a standalone H-NOX from *Nostoc sp.* PCC 7120 (33% identity, PDB: 2o09). The representative PAS domain is from *Nostoc punctiforme* histidine kinase (35% identity, PDB: 2P04). The helical domain (PDB: 3HLS) and catalytic domain (PDB: 3UVJ) are crystallized truncations of sGC from *Rattus norvegicus* and *Homo sapiens*, respectively.

## Conclusion

Together these approaches define the architecture of the sGC holoenzyme, revealing inter-domain interactions responsible for communicating NO-occupancy from the heme to the catalytic site. The resultant structural model of sGC provides insight into the mechanisms of activation of both NO and small molecule modulators.
